# Country-Specific Conditions for Work and Family Reconciliation: An Attempt at Quantification

**DOI:** 10.1007/s10680-015-9366-9

**Published:** 2016-01-28

**Authors:** Anna Matysiak, Dorota Węziak-Białowolska

**Affiliations:** 1 Wittgenstein Centre for Demography and Global Human Capital, Vienna Institute of Demography, Austrian Academy of Sciences, Welthandelsplatz 2, 1020 Vienna, Austria; 2 Institute of Statistics and Demography, Warsaw School of Economics, Ul. Madalińskiego 6/8, 02-513 Warsaw, Poland; 3 Econometrics and Applied Statistics Unit, Deputy Directorate General, European Commission Joint Research Centre, Via E. Fermi 2749, TP 361, 21027 Ispra, VA Italy

**Keywords:** Family policies, Fertility, Gender norms, Labour market structures, Uncertainty and sensitivity analysis

## Abstract

The country-specific conditions for work and family reconciliation (family policies, labour market structures and gender norms) are believed to influence tensions between paid employment and childbearing. So far there have been very few attempts to quantify these conditions into a single measure which would allow for comparisons across countries of the magnitude of the barriers that working parents encounter. Such a quantitative index could also facilitate a quantitative investigation of the association between the macro-level conditions for work and family reconciliation and fertility at the individual level. In this paper, we seek to fill this gap by proposing a quantitative index of country-specific conditions for work and family reconciliation, which may be used, for example, in a two-level regression framework. The index takes into account all three components of the conditions for work and family reconciliation. We also perform a series of uncertainty and sensitivity analyses which verify the robustness of our assumptions and which illustrate the range of the index volatility.

## Introduction

Since the onset of industrialization, childrearing and participating in the labour market has become increasingly incompatible. Work places have become separated and distant from people’s homes, the work schedules are inflexible, and the increasing pressure on employees regarding time availability, mobility and skill improvement adds to the difficulties faced by parents struggling to combine paid work with family life (Brewster and Rindfuss 2000; Goldscheider et al. 2015). Nonetheless, it has been widely argued in the demographic and sociological literature that the conditions for work and family reconciliation (CWFR), in which couples make their decisions about childbearing and paid employment, are substantially better in countries where (1) family policies support parents in combining paid work with childcare (Esping-Andersen 2009), (2) labour market laws allow for more flexible organization of the working time and eliminate barriers to employing non-incumbent workers (Adserà 2004, 2005) and (3) gender norms are more egalitarian (Liefbroer and Corijn 1999). In those countries, tensions between paid employment and childbearing are supposed to be weaker, and as a consequence, mothers may be more present and more successful in the labour market (Pettit and Hook 2005; Misra et al. 2011). Furthermore, it might be easier for women with occupational ambitions to decide to have a/another child (McDonald 2000; Esping-Andersen and Billari 2015). All in all, better CWFR may translate into higher fertility and/or higher female employment (Ahn and Mira 2002; Rindfuss and Brewster 1996; Engelhardt et al. 2004).

Because of the importance of country-specific CWFR for fertility, and because these conditions are highly relevant to policy decisions, many attempts have been made to describe and assess the CWFR in advanced economies. These attempts generally consisted of a detailed analysis of certain single dimensions of these CWFR, most often family policies, upon which a classification of countries was proposed. Based on the results of these analyses, various family policy regimes (e.g. Anttonen and Sipila 1996; Gornick et al. 1997; Trifiletti 1999; Korpi 2000; Bettio and Plantenga 2004; Thévenon 2011) and gender role attitudinal regimes (Treas and Widmer 2000; Lück and Hofäcker 2003; Philipov 2008) were suggested. These typologies proved useful in providing us with information about the general ideology underpinning family policy and attitudinal regimes, yet failed to inform us about the absolute magnitude of the barriers experienced by parents in combining work and family in a given country, or about the relative standing of a country in the area of work and family reconciliation. This kind of information can only be provided by a quantitative indicator—which would also be useful to researchers conducting investigations of the associations between country-specific CWFR and various aspects of individual lives, including childbearing or mothers’ subjective well-being (Schober and Schmitt 2013; Aassve et al. 2014).

Despite the obvious benefits of compiling a quantitative index assessing country-specific CWFR, to the best of our knowledge there have been very few attempts to propose such a measure. Gornick et al. (1997) and Gornick and Meyers (2003) are among the first scholars to advance this idea. Their index of public support of employment for mothers measured the level of public support provided to working mothers and thus allowed them to construct a country ranking of these levels. Ray et al. (2010) went a step further and proposed the Gender Equality Index which measures not only generosity but also gender equality in public provisions. A more general index measuring the degrees of incompatibility between work and family, proposed by Matysiak (2011), took into account not only family policies but also, in contrast to previous studies, labour market structures and gender norms. However, all these attempts suffer from the assumption that the overall index responds in the same way to the change in one of its components irrespective of their initial values as well as of the level of other components. Moreover, they disregard correlations between the index components, which leads to a situation in which correlated components are of higher importance for determining the final index.

In this paper, we expand the previous study by Matysiak (2011). First, we develop a conceptual model of the country-specific CWFR. Second, we summarize this model into a quantitative index of conditions for work and family reconciliation (ICWFR). Compared to the index proposed by Matysiak (2011), the ICWFR uses more recent data and a wider set of indicators, taking the methodological shortcomings of the previous indices into account in the aggregation process. Furthermore, it is tested in two ways: for robustness with respect to methodological normative assumptions made in the process of its construction in a series of uncertainty and sensitivity analyses and for criterion validity with respect to fertility and the labour force participation of mothers, which are the external criteria theoretically predicted to positively correlate with the ICWFR.

## Country-Specific Conditions for Work and Family Reconciliation: A Conceptualization

Demographic and socio-economic literature has identified three main groups of macro factors affecting the CWFR: (1) family policies, such as public childcare provision and parental leave mandates for women and men (e.g. Gauthier 1996; Esping-Andersen 2009); (2) labour market structures, including the flexibility of working hours and employment protection legislation that affect the costs of firing and hiring (e.g. Ahn and Mira 2002; Adserà 2004, 2005); and (3) social norms regarding men’s and women’s roles (e.g. Liefbroer and Corijn 1999; Muszyńska 2007). We briefly present below the major theoretical arguments for the effects of these three groups of factors on fertility and women’s labour supply. Next, we propose a conceptual scheme of the CWFR.

### Family Policies

Among family policies, the main instruments designed to support work and family reconciliation are childcare services and parental childcare leaves for both mothers and fathers. They constitute an important dimension of *family policy*-*related CWFR*.

#### Childcare Services

Childcare services are among the most important instruments facilitating reconciliation of paid work and family life. First, an improvement in childcare provision reduces the opportunity costs of parenting, which should lead to an increase in the demand for children. Second, better childcare provision leads to a reduction in the mothers’ reservation wage and thus is expected to encourage women to return to the labour market earlier. Childcare provision, which facilitates reconciliation of paid work and family, should generally be well accessible in terms of the number of available places, openings hours and costs but should also be of a high quality to constitute a reasonable alternative to parental care (Plantenga and Remery 2009).

The positive impact of childcare accessibility on women’s employment is widely documented in the literature regardless of whether studies looked into childcare supply or childcare costs (e.g. Connelly 1992; Kimmel 1995; Del Boca 2002; De Henau et al. 2011; Pettit and Hook 2005; Misra et al. 2011). The research on the impact of childcare quality on women’s employment is very limited, but the available evidence for low-income mothers suggests that higher-quality childcare facilitates their return to paid work and improves their employment stability (Benasich et al. 1992; Meyers 1993).

The effect of childcare provision on fertility seems to be more mixed. Del Boca (2002) found that childcare availability has a positive impact on fertility, whereas Hank and Kreyenfeld (2003) and Andersson et al. (2004) found that it has no significant effect. One problem with these studies is, however, that they treat childcare availability as exogenous to fertility. The newest studies, which account for this problem, have shown unequivocally that having access to public childcare facilitates childbearing (Baizan 2009; Rindfuss et al. 2010). It should be noted, however, that empirical studies usually look at childcare supply in terms of the number of available places, disregarding other important childcare characteristics such as opening hours, costs or quality of the services.

#### Childcare Leaves

There are various types of leave that may be claimed by parents: maternity leave directed at mothers; paternity leave directed at fathers; parental leave directed at both parents, although usually used by women; the “daddy quota”, which is the portion of the parental leave entitlement reserved exclusively for fathers; and sick-child leaves which are usually short-term leaves that allow parents to take care of a sick child.

The impact of maternity and parental leave entitlements on women’s employment depends on the length of the leave and on the related benefit. Generally, well paid but short leave entitlements are considered to have a positive impact on women’s employment levels as they increase women’s attachment to the labour market, without evoking a negative impact on their human capital (Thévenon and Solaz 2014). Longer leaves, however, may decrease women’s employment and earning prospects (Ruhm 1999). Empirical research has generally confirmed the non-monotonic relationship between leave duration and women’s employment (Pettit and Hook 2005; Baker and Milligan 2008; De Henau et al. 2011), but there is no agreement about the optimal duration of parental leave (Galtry and Callister 2005). Some studies show that only short—4–5 months long—child-related career breaks (corresponding in practice to maternity leave) are not detrimental to a woman’s employment career (Baker and Milligan 2008; Evertsson and Duvander 2011), while others suggest that even longer leaves of 1.5–2 years do not jeopardize women’s careers (Misra et al. 2011; Thévenon and Solaz 2014).

As maternity and parental leave provisions are expected to reduce the opportunity costs of parenting, they are expected to stimulate fertility. In general, empirical research has shown that childbearing decisions are mainly affected by the amount of compensation and the payment conditions of the parental leave benefit (Thévenon and Gauthier 2011). The clearest examples of the positive effects of parental leave benefits on fertility were found in Sweden (Hoem 1993; Andersson 1999) and Austria (Lalive and Zweimueller 2009), where speed premium systems were built into the parental leave schemes. The introduction of parental allowances was also shown to have a positive influence on fertility in Finland, Norway and France, mainly with respect to third births (Vikat 2004; Aassve and Lappegård 2009).

Compared to fertility and employment effects of leave uptake among women, we know much less about the effects of leave entitlements directed at men. In general, greater involvement of men in childcare is expected to reduce the care burden on women and thus facilitate fertility and/or women’s return to the labour market (McDonald 2000; Esping-Andersen 2009). The few studies on the impact of parental leave uptake by men on fertility, which were conducted in the Scandinavian context, have provided evidence consistent with this expectation (Duvander et al. 2010). Moreover, a father’s use of the leave was demonstrated to be positively related to his later involvement in childcare, thus reducing the care burden for women (Haas and Hwang 2008; Seward et al. 2006). However, the obtained effects do not appear to be causal, as these studies did not account for a selection of family-oriented fathers into the use of parental leave.

Apart from regular employment interruptions for taking care of a young child, parents of children in all age groups may also need to take some time off from work because of short-term needs of their children, such as child’s sickness, the need to undergo a routine preventive medical check-up or a breakdown of childcare arrangements. Such unpredicted events were shown to induce high levels of stress on working parents (Galinsky and Stein 1990; Zedeck and Mosier 1990). In this light, paid sick-child leaves should lessen the tensions experienced by working parents (Gornick and Meyers 2003; Waldfogel and McLanahan 2011). Indeed, such a finding was obtained by Allen et al. (2014) for married parents in 12 OECD countries.

### Labour Market Structures

Two characteristics of labour markets, the flexibility of work arrangements and the magnitude of the barriers to labour market entry, have been claimed to be important determinants of women’s fertility and labour market behaviours (Adserà 2004, 2005, 2011; Ahn and Mira 2002). By influencing women’s opportunities to enter the labour market, maintain employment and combine work with family duties, these characteristics define the extent to which the labour market has adjusted to accommodate female labour—in other words, they determine the country-specific *labour market*-*related CWFR*.

#### Flexibility of Working Hours

Reducing the working hours by moving from full-time to part-time employment is one of the most common options for a parent who wishes to continue economic activity but for whom a full-time working schedule is not compatible with childrearing responsibilities (Hegewisch and Gornick 2011). In fact, establishing part-time work opportunities has greatly contributed to an increase in women’s labour force participation in many developed countries (Del Boca 2002; Jaumotte 2003; Aaberge et al. 2005). However, this form of employment often comes at a cost, as it may lower women’s career prospects in terms of hourly wages and attainable positions (Walsh 2007; Hegewisch and Gornick 2011; O’Reilly and Bothfeld 2002); it may also lead to employment at non-standard hours (Gornick and Heron 2006).

Since part-time employment reduces the tensions between paid work and family, it may encourage mothers to enlarge their families. In fact, some studies found positive effects of part-time work on birth risks (e.g. Berkowitz King 2005; Liefbroer 2005; Schmitt 2012), but there are also studies that did not yield any significant effects (e.g. Kreyenfeld 2005; Oláh 2003; Schmitt 2012). This lack of positive findings may be related to the fact that occupational downgrading resulting from part-time employment may discourage women from childbearing. Mothers working part-time who perceive their earning, advancement or training opportunities to be worse than those of full-time employees may try to return to full-time employment as soon as possible, which may keep them from enlarging their families. Consistent with these arguments, it was shown that the effects of part-time employment on fertility are positive once the part-time jobs are of a high quality (Del Boca et al. 2009).

However, work flexibility can be achieved not only through part-time employment but also by granting parents more control over their working lives, including the hours of work and the location where the work is performed. Studies have demonstrated that giving parents the option of adjusting their working hours in order to meet their family obligations (e.g. Byron 2005; Allen and Shockley 2009; Hill et al. 2010) or of working from home (Gajendran and Harrison 2007) reduces the work–family conflict.

#### Barriers to Labour Market Entry

The barriers to labour market entry are highest in countries characterized by strong employment protection, which secures the positions of the permanently employed but hinders access to secure work for unemployed and temporarily employed outsiders (OECD 2004). Consequently, employment protection has a positive influence on the employment opportunities of prime-aged men, who are largely employed, but lowers the employment prospects of labour market entrants—or of individuals who take temporary employment breaks, i.e. women (Bertola et al. 2007; Kahn 2007).

Only recently barriers to labour market entry for women and young people have been linked to low fertility (Del Boca 2002; Adserà 2004, 2005; Aaberge et al. 2005). It was argued that by hindering women from returning to paid employment after a child-related career break, employment protection affects the opportunity costs of parenting and thus prevents women from realizing their fertility intentions (Adserà 2004, 2005; Aaberge et al. 2005). In this vein, Adserà (2004) and Rovny (2011) showed that strong employment protection legislation is indeed negatively related to total fertility; Fogli (2004) demonstrated that it leads young adults to postpone their leaving the parental home and Adserà (2005, 2011) provided evidence that high gender unemployment gaps and long-term unemployment produced by the insider/outsider divide substantially slow down the progression to third births.

### Gender Norms

In addition to family policies and labour market structures, conditions for work/family reconciliation are also influenced by broad ideologies and norms regarding the “correct” division of unpaid household work and paid market labour between women and men that dominate in a given society (Pfau-Effinger 2000; Arpino et al. 2015; Aassve et al. 2014). These society-level norms influence women’s and men’s individual decisions about the adopted division of labour and also shape employers’ perceptions and their treatment of male and female workers, thereby affecting the opportunity costs of parenting and working for pay. In countries where the traditional division of labour is more ingrained, the opportunity costs are higher as women feel expected to withdraw from employment after they become mothers and fathers are more discouraged from taking child-related career breaks than in countries with more egalitarian beliefs (Budig et al. 2012). In the former societies, women may also tend to postpone parenthood decisions or even abandon having children if the gender norms dominant in the country clash with their individual beliefs (Liefbroer and Corijn 1999; Muszyńska 2007). We will use the country-specific *culture*-*related CWFR* when referring to the conditions caused by gender norms.

Empirical studies have generally shown that more egalitarian gender norms at the society level are positively linked with women’s labour market outcomes in terms of employment (Fortin 2005; Algan and Cahuc 2007) and amplify the positive effect of reconciliation policies on women’s wages (Budig et al. 2012). It should, however, also be noted that Uunk et al. (2005) found that the positive effect of egalitarian gender norms on women’s working hours disappeared after controlling for family policies. Social acceptance of mothers’ employment as well as fathers’ involvement in childcare has also been widely argued to create good conditions for childbearing in modern societies where women aspire for high education and occupational careers (Goldscheider et al. 2014; Esping-Andersen and Billari 2015). Consistent with this view, empirical studies that examine society-level gender norms find that egalitarian gender norms facilitate family formation (Gimenez-Nadal et al. 2012; Sevilla-Sanz 2010).

### A Conceptual Scheme

**Fig. 1:**
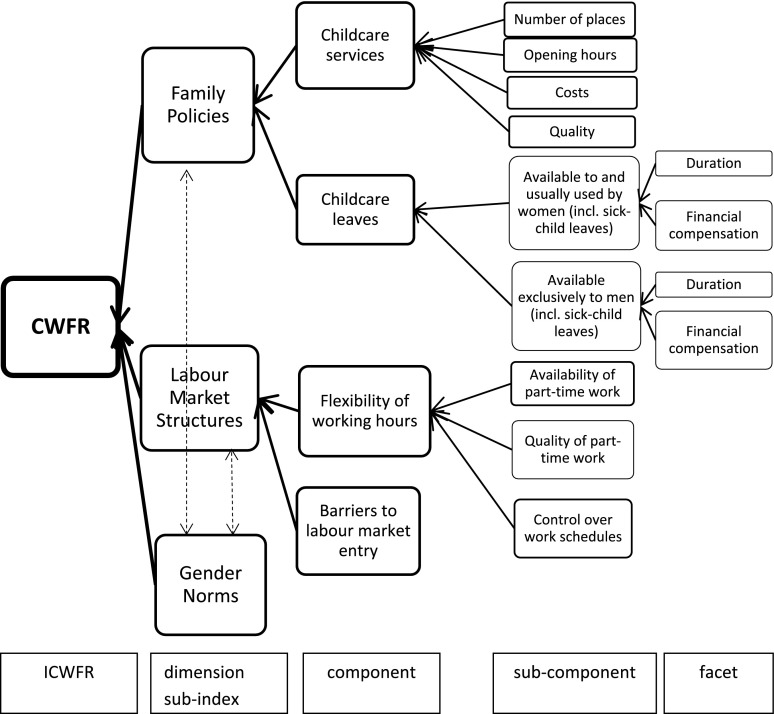
Conceptual scheme of the country-specific conditions for work and family reconciliation (CWFR)

Overall, we see the CWFR as a product of three dimensions: family policies, labour market structures and gender norms (see Fig. 1). Of the family policies, we consider childcare services (in terms of number of places, quality, opening hours and costs) as well as childcare leaves for both women and men (in terms of duration and financial compensation), including nursing leaves. The labour market dimension of the CWFR covers flexible working hours, such as opportunities to work part-time and the quality of part-time jobs as well as having control over one’s work schedule, but also barriers to labour market entry, usually caused by employment protection regulations. Finally, culture-related CWFR encompass gender norms.

This conceptual scheme constitutes a basis for the construction of the ICWFR. The index is composed of three sub-indices: the family policy index (FPI), the labour market structure index (LMSI) and the gender norms index (GNI), each of which is constructed using certain components and sub-components presented in Fig. 1. Given this structure, we claim that the ICWFR has a formative nature (Bagozzi 2007; Bollen 2007; Howell et al. 2007), which implies that the CWFR are determined by (and not reflected in) particular policies and social norms.

Several important assumptions must be made at this stage of the construction of the ICWFR. The first is with regard to the compensability of the elements of the scheme. Can a country’s poor performance in one of the CWFR dimensions be fully compensated for by a good performance in another dimension? Although there are no specific studies on this topic, we argue that assuming partial instead of full compensation is more accurate. Partial compensation means, for example, that implementing work/family reconciliation policies in a country with traditional gender norms may not have the same effects as in a country with egalitarian gender norms, as individuals who adhere to a family model based on role specialization may be less likely to take advantage of reconciliation measures.

The sensitivity of the ICWFR to changes in its dimensions, components and sub-components is the second issue that requires consideration. For example, does an expansion of childcare services improve the conditions of working parents more in countries where these services are poor, or in countries where they are already well developed? Although there are no indications on this issue in the literature, in our opinion the improvement in the CWFR should be larger in countries where the shortage in childcare services is larger. Thus, we assume that the relationship between the ICWFR and its dimensions, components and sub-components is nonlinear, favouring greater improvements in those index components which are underdeveloped.

Finally, are the three dimensions of the CWFR equally important? Likewise, are the components of each dimension equally important? To the best of our knowledge, there has been no research yet on the relative importance of the various factors affecting the CWFR, which could guide us in establishing the aggregation weights. In this paper, we thus assume that family policies, labour market structures and gender norms should contribute evenly to the CWFR, as we have no grounds to assume diverse importance. Similarly, components are assumed to be equally important in determining each dimension, and sub-components in determining each component.

## Data

The main criteria that guided our search for indicators were the relevance of the indicator for the concept measured but also the reliability of its data source, its timeliness and country coverage. The indicators refer to the period between 2008 and 2010. Their final list together with the associated data sources, the time period covered and the relationship of each indicator to the ICWFR is presented in Table 1, and the data are available upon request.

**Table 1:** List of variables used for ICWFR construction

Component	Variable name	Description (orientation towards ICWFR)	Time period	Source
Family policies dimension				
Childcare services	CHHOURS_03	Average number of hours spent in formal childcare by children below three (children with or without formal childcare are taken into regard in the denominator—[ilc_camnforg0]) (positive)	2009	EU-SILC data available online in Eurostat
	CHHOURS_35	Average number of hours spent in formal childcare by children aged three to compulsory school age (children with or without formal childcare are taken into regard in the denominator [ilc_camnforg0]) (positive)	2009	EU-SILC data available online in Eurostat
	CHHOURS_6+	Average number of hours spent in formal childcare by children in compulsory school age (children with or without formal childcare are taken into regard in the denominator [ilc_camnforg0]) (positive)	2009	EU-SILC data available online in Eurostat
	CHQUALITY	Children-to-staff ratio in childcare institutions (positive)	2008/2009	OECD Family Policy Database/Plantenga and Remery (2009)
	CHCOST	% out-of-pocket expenses on childcare and the net income of dual-earner family with each partner earning the average salary in the national economy (negative)	2008	OECD Tax and Benefits Database
Childcare leaves	MLEAVE	Maternity and parental leave available for mothers in the first year after birth in full-time equivalents (i.e. leave duration in the first year after birth multiplied by the income replacement rate of the respective leave benefit) (positive)^a^	2009	Multilinks supplemented with information from Moss (2009)
	FLEAVE	Paternity and parental leave reserved for fathers in full-time equivalents (positive)	2009	Multilinks supplemented with information from Moss (2009)
	SLEAVE	Sick-child leave in full-time equivalents per parent (positive)	2009	Council of Europe Family Policy Database
Labour market structures dimension				
Flexibility of working hours	FWSCHED	% employees with flexible work arrangements (i.e. employees who could determine their own work schedule or worked in companies with working time banking or companies with daily fixed number of working hours but flexibility as to their use during the day) among women aged 25–49 [lfso_l0fvar] (positive)	2010	Ad hoc module to Labour Force Survey in 2010, data available online in Euro stat
	PART_AVAILABILITY	Part-time employment as percentage of the total employment of women aged 25–49 (%) [lfsi_emp_a] (positive)	2009	Labour Force Survey data available online in Euro stat
	PART_QUALITY	Ratio of hourly wages in part-time to full-time employment, women aged 25–49 (positive)	2009	Authors’ own computations based on the EU-SILC data
Barriers to labour market entry	EPL	Indicator of overall employment protection legislation for regular contracts EPR_vl (negative)	2009	Avdagic (2012) for post-socialist countries of Europe, OECD (2013) for remaining countries
Gender norm dimension				
Components to be extracted at the stage of exploration and verification of the data structure	GN1	% of people who agree or strongly agree with the statement that a working mother can establish just as warm and secure a relationship with her child as a mother who does not work [VI59] (positive)	2008	European Value Study
	GN2	% of people who disagree or strongly disagree with the statement that a preschool child is likely to suffer if his or her mother works [VI60] (positive)	2008	European Value Survey
	GN3	% of people who disagree or strongly disagree with the statement that a job is all right, but what most women really want is a home and children [VI61] (positive)	2008	European Value Survey
	GN4	% of people who agree or strongly agree with the statement that, in general, fathers are as well suited to look after their children as mothers [VI65] (positive)	2008	European Value Survey
	GN5	% of people who agree or strongly agree with the statement that men should take as much responsibility as women for the home and children [VI66] (positive)	2008	European Value Survey

^a^The part of the parental leave reserved for fathers is not included

Several data sources were used to quantify family policy-related CWFR. Childcare availability in terms of number of places and opening hours was measured with the average number of hours spent in formal childcare by children aged below three, children aged three to compulsory school age and children of compulsory school age (CHHOURS_03, CHHOURS 36, CHHOURS_6+). These averages, derived from the European Union Survey on Income and Living Conditions (EU-SILC) data, were computed after including into the denominator all children, namely both those attends and those not attending formal childcare. It is noteworthy that measuring access to childcare by number of available places and their opening hours could seem to be more appropriate than using data on enrolment. The reason is that, to the best of our knowledge, such data are not available for children of school age for all countries covered by our analysis. Dropping the information on childcare availability for children in this age group would, in our view, constitute a greater loss of information than replacing data on childcare supply with data on enrolment given that an excessive supply of childcare places will usually adjust downward with time to the actual childcare demand.

The quality of childcare (CHQUALITY) was assessed with a children-to-staff ratio available in the OECD Family Policy Database and in Plantenga and Remery (2009). The children-to-staff ratio for most countries covered by the database was available separately for institutions for children below and above age 3, but for some countries only an overall ratio was given. In order to cover the maximum number of countries, we computed the overall ratio for all countries by weighting the ratios by children’s age with the proportions of children in a given age group out of all children attending childcare institutions. The costs of childcare (CHCOST) were measured with a ratio of out-of-pocket expenses on childcare and the net income of a dual-earner family with each partner earning the average salary in the national economy. The out-of-pocket expenses are calculated by the OECD as the difference in net income of a family making use of formal childcare and an otherwise identical family not using such childcare. Hence, the measure takes into account public subsidies on childcare as well as an interaction between childcare policies and tax and benefit policies (the OECD Tax and Benefits Database).

When it comes to childcare leaves, we first had to make a decision on the duration of the parental leave that could be considered as a part of the ICWFR, i.e. the duration that reduces tensions between paid work and childrearing without jeopardizing women’s future employment prospects. Due to the lack of consistent suggestions in the literature on this topic, we decided to consider the parental leave entitlements in the first year after birth as a key parameter determining the CWFR. Time spent with parents in the first year of a child’s life is the most crucial for the well-being, cognitive and emotional development of the child (Brooks-Gunn et al. 2010; Joshi et al. 2009), and childcare opportunities for children below age 1 can be very limited in many countries (authors’ calculations based on the EU-SILC available on request). Hence, parents may have strong motivations to interrupt their employment careers during the first year of a child’s life and may experience a particularly strong work–family conflict if they are not eligible for paid leave during this time.

Data on childcare leaves were extracted from the Multilinks Database on Intergenerational Policy Indicators for Family Policies (Keck et al. 2009) and supplemented with data available in Moss (2009), which provided us with data on the duration of maternity, paternity and parental leaves (including daddy quotas) as well as financial compensations paid during such leave relative to pre-birth earnings. In some countries, compensation rates depend on the length of the leave taken. In those cases, we used the compensation rate equal to the rate paid if for a leave of no longer than 1 year. Using these data, we computed duration of paternity and parental leaves in full-time equivalents available exclusively to men (FLEAVE) and the number of months of maternity and parental leave in full-time equivalents available to and usually used by women in the first year after birth (MLEAVE). Finally, the data on sick-child leaves were extracted from the Council of Europe Family Policy Database and it was used to compute duration of sick-child leaves in full-time equivalents per parent (SLEAVE).

Moving to the labour market structures dimension, we assessed the availability of part-time jobs with the proportion of women aged 25–49 working part-time available from the European Labour Force Survey (PART_AVAILABILITY). This variable refers to outcomes of work and family reconciliation rather than conditions, but to the best of our knowledge no better measure of the supply of part-time jobs is available. Furthermore, this variable, in our view, can serve quite well as a proxy of the availability of part-time jobs and hence capture the CWFR. Even though in countries with low provision of childcare women may be forced into part-time employment, their CWFR will still be better if they are offered a possibility to reduce working hours than for women who do not even have that option. For instance, while women in Germany or the Netherlands, where external childcare options are limited, can easily switch to part-time employment after birth, in the central and eastern Europe women often face a rigid choice between full-time work or no work at all, and in that respect the latter face worse conditions for combining paid work and childcare than the former (Pascall and Kwak 2005; Tang and Cousins 2005; Cazes and Nesporova 2004).

Apart from the availability of part-time jobs, we also take into account their quality. The quality of part-time jobs was evaluated on the basis of the EU-SILC data with a ratio of hourly wages earned in part-time and full-time jobs by female employees aged 25–49 (PART_QUALITY). Such a ratio is commonly used for assessing the quality of part-time employment (Burgess 2005; Chalmers et al. 2005; Del Boca et al. 2009). For measuring control over work schedule, we used the proportion of employees with flexible work arrangements, i.e. employees who could determine their own work schedule or worked in companies with working time banking or companies with daily fixed numbers of working hours but flexibility as to their use during the day (FWSCHED). Such data were collected in the ad hoc module to the Labour Force Survey in 2010 on reconciliation between work and family. The barriers to labour market entry were measured with the index of employment protection legislation for regular contracts (EPR) developed and computed for OECD countries by the OECD (2013). For central and eastern European countries, we used the EPR computed by Avdagic (2012) and Avdagic and Salardi (2013) for countries from this region using the OECD methodology.

Finally, information on gender norms was obtained from the European Value Survey 2008 which asks a battery of questions on attitudes towards the involvement of women and men in paid employment, family care and household chores (GN1–GN5). For each statement, we computed the proportion of persons agreeing or strongly agreeing with the statement if it expressed a social support for egalitarian division of labour among women and men and the proportion of persons disagreeing and strongly disagreeing with the statement in the opposite case.

Given the available data, we succeeded in covering 30 countries: 27 European Union (EU) member states as well as Iceland, Norway and Switzerland. This was the largest set of countries for which we were able to retrieve comparable indicators relevant to the measurement of the CWFR. However, even in this data set 13 missing values were observed. They related to Malta (three cases), Cyprus, Romania, Switzerland (two), Iceland, Italy, Latvia and Luxembourg (one). Looking from the variable perspective, seven missing entries were observed for two variables from family policies dimension (four times for childcare quality and three times for childcare cost variables) and six for labour market structures dimension (twice for each variable but PART__AVAILABILITY). To impute them, we used the expectation–maximization (EM) algorithm (Little and Rubin 2002) which is a very effective method when the data are correlated, such as in our case.

## Methods

The construction of a composite indicator is not a straightforward procedure as it involves assumptions that have to be assessed carefully; otherwise, the final product will be of dubious analytic rigour (Saltelli 2006). Although these methodological aspects had our special attention, in this and the following sections we present only the most crucial issues. Nevertheless, all details concerning the construction of the ICWFR are available from the authors upon request.

First, we performed an operationalization of our conceptual model of the CWFR by verifying the underlying structure of our data. Verification was conducted using the principal component analysis (PCA). Our criteria for component extraction were based on the eigenvalue level (Kaiser criterion), the amount of variance explained and the pattern of principal component (PC) loadings. It is noteworthy that we used PCA only to confirm our conceptual model, and not for computing the scores for sub-components, as doing so would mean that we had accepted the full compensability among variables.

**Fig. 2:**
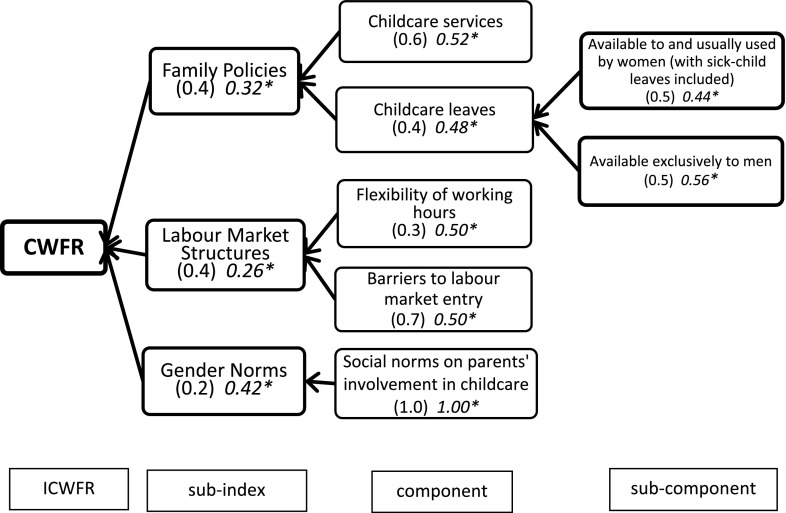
Operationalization scheme of the ICWFR. *Note* Approximate importance of the components and sub-components of CWFR is given in *italics* and with *asterisk*. The resulting aggregation weights are given in *brackets*. Aggregation weights attributed to the variables populating the sub-components/components (not presented) are always equal

Second, to aggregate variables, sub-components, components and sub-indices (see Fig. 2) into the ICWFR, we employed a generalized mean with power *q* = 0.5, which is between the arithmetic mean (a generalized mean with a power equal to one) and the geometric mean (a generalized mean with a power equal to zero). In contrast to the arithmetic mean, which was used in previous studies attempting to quantify the CWFR [see Gornick et al. (1997), Matysiak (2011)], this aggregation technique ensures that there is no possibility of a full compensation of low results in one component or dimension with high results in others (Decancq and Lugo 2013; Ruiz 2011). It also ensures that a rise in the lower tail of distribution of any variable will improve the composite indicator more than a similar increase in the upper tail (Ruiz 2011). Such an approach is in line with the assumptions of our conceptual model on partial incompensability and nonlinear relationship between the change in the index and its sub-indices, components and sub-components. It also corresponds to recent developments in the field; it was used for computing the Human Development Index (HDI) as of 2010 (Klugman et al. 2011) and the Material Condition Index proposed by Ruiz (2011) for the OECD. The influence of the power of the generalized mean on the results was verified using uncertainty and sensitivity analyses.

The aggregation was performed on normalized variables (1–100), which also resulted in obtaining the ICWFR expressed in normalized values. In the aggregation process, we aimed at ensuring that equal importance was given to each sub-component, component and dimension for the ICWFR. To this end, we established the aggregation weights according to the approach proposed by Paruolo et al. (2013). Paruolo et al. (2013) showed that applying an equal-weighting scheme may lead to the situation where more importance in determining the composite indicator is given to those components which are correlated and have higher variances. They proposed a method which allows to establish the aggregation weights after taking the covariance matrix of the index components into account.^1^

Finally, we performed the uncertainty analysis and sensitivity analysis in order to assess the robustness of the ICWFR with regard to all the normative assumptions made during the conceptualization process, namely assumptions regarding the non-full compensability, the nonlinear relationship between the index and its components, the method of imputation and the weighting (see Sect. 2.4). The aim of the uncertainty analysis was to present the overall possible variation in the ICWFR scores resulting from the uncertainty linked to the assumptions made. The aim of the sensitivity analysis was to determine which of the assumptions had the most influence on the scores.

In order to verify the assumption regarding the rate of non-full compensability and the strength of the nonlinear relationship between the index and its components, we modified the power of the generalized mean, which was allowed to range from <0.2; 1>. The assumption on weighting was tested by assuming that weights at the sub-index level range ±5 % of the reference weight, and the assumption on the imputation method by allowing the hot-deck imputation^2^ next to the EM.

The three uncertain factors—the power of the generalized mean, the weights and the imputation method—were sampled simultaneously in a quasi-random sampling scheme (Sobol 1976), with a base sample of *n* = 3000 in order to capture all of the possible interaction effects among the assumptions made. In the uncertainty analysis, the simulated indices were compared with the reference index. The final score is therefore presented with the uncertainty expressed by the error terms. In the sensitivity analysis, we used Sobol’s sensitivity indices: the first-order effect *S*_*i*_ (Saltelli et al. 2000; Sobol 1993) and the total effect $S_{{{\text{T}}_{\text{i}} }}$ (Saltelli et al. 2010; Homma and Saltelli 1996).^3^ The first-order effects *S*_i_ tell us what proportion of the variance in the ICWFR was caused by the uncertainty factors. Since these indices do not take interactions involving the uncertainty factors into account, we also computed the total effects $S_{{{\text{T}}_{\text{i}} }} ,$ which tell us about the overall influence of the uncertainty factors on the composite, including the interactions (Homma and Saltelli 1996). We considered the uncertainty factor to have an important influence on the composite indicator if it explained at least 1/*n* × 100 % of the variance in the composite indicator, where *n* is a number of uncertain factors (Saisana et al. 2005).

## Results

### Operationalization Scheme

According to our conceptualization scheme presented in Fig. 1, the family policies dimension consists of two components: childcare services and childcare leaves. The structure of both components was confirmed in our data. For the childcare services, we obtained a one-dimensional solution (Table 2) explaining 52.1 % of the variance present in the data. For the Childcare leaves, as expected we obtained a two-dimensional solution with maternity and parental leaves available to women (MLEAVE) as well as sick-child leaves (SLEAVE) loading to the first PC and leaves available exclusively to men (FLEAVE) loading to the second PC (Table 3). Sick-child leaves were found to group together with maternity and parental leaves available to women likely because they are usually taken by women. The variance explained accounted for 56.3 and 33.8 % (summed 90.1 %) for the first and second PC, respectively.

**Table 2:** Pattern PC loadings in the one-dimensional PCA for the childcare services component of the family policy index (only loadings above 0.4 are reported)

Variable	Childcare services
CHHOURS_03	0.838
CHHOURS_36	0.879
CHHOURS_6+	0.791
CHQUALITY	0.447
CHCOST	−0.548

**Table 3:** Pattern PC loadings in the two-dimensional PCA for the childcare leaves component of the family policy index (only loadings above 0.4 are reported)

Variable	Leaves for women	Leaves for men
MLEAVE	0.913	
SLEAVE	0.922	
FLEAVE		0.994

Conceptually, the labour market structures dimension consists of two components: (1) the flexibility of working hours and (2) barriers to labour market entry. This conceptualization was confirmed by the PCA. Two PCs were extracted. The first PC, corresponding to the flexibility of working hours, was loaded by three variables: the proportion of women in part-time employment (PART_AVAILABILITY), the quality of part-time jobs (PART_QUALITY) and the proportion of women with flexible work schedules (FWSCHED) (see Table 4). The second PC, corresponding to barriers to labour market entry, was loaded by the EPR variable. The variance explained accounted for 46.6 and 23.2 % (summed 69.8 %) for the first and second PC, respectively.

**Table 4:** Pattern of PC loadings in the two-dimensional PCA solution for labour market structures index (only factor loadings above 0.4 are reported)

Variable	Flexibility of working hours	Barriers to labour market entry
FWSCHED	0.843	
PART_AVAILABILITY	0.728	
PART_QUALITY	0.682	
EPR		0.907

The gender norms dimension has no conceptual scheme. In this case, we performed an exploratory analysis on all five indicators, populating this dimension to reveal its components. It appeared that only one eigenvalue exceeded 1 and that the level of explained variance by the first PC amounted to 63.7 %. This means that the values assumed by the GN1–GN5 variables were driven by one latent variable that describes social norms regarding parents’ involvement in childcare, including social norms regarding women’s participation in paid employment, women’s participation in childcare and men’s participation in childcare (see Table 5).

**Table 5:** Pattern of PC loadings in the one-dimensional PCA solution for the gender norms index (only factor loadings above 0.4 are reported)

Variable	Social norms on parents’ involvement in childcare
GN1	0.787
GN2	0.901
GN3	0.763
GN4	0.840
GN5	0.681

Overall, the multivariate analyses we performed led us to confirming the conceptualization scheme presented in Fig. 1, except for the assignment of sick-child leaves. The final operationalization scheme, used to compute the ICWFR, is presented in Fig. 2. This figure also contains information about the assumed importance of the components and dimensions for determining the CWFR (in italics with an asterisk) as well as the resulting aggregation weights (in brackets). As can be seen, the GNI is weighted with a weight 0.2 only, but its importance for determining the ICWFR is relatively large due to the correlation of GNI with FPI and because of its relatively large variance. Applying larger weights to the GNI would further increase its importance, which would not be consistent with our conceptualization scheme.

### Index Scores

**Fig. 3:**
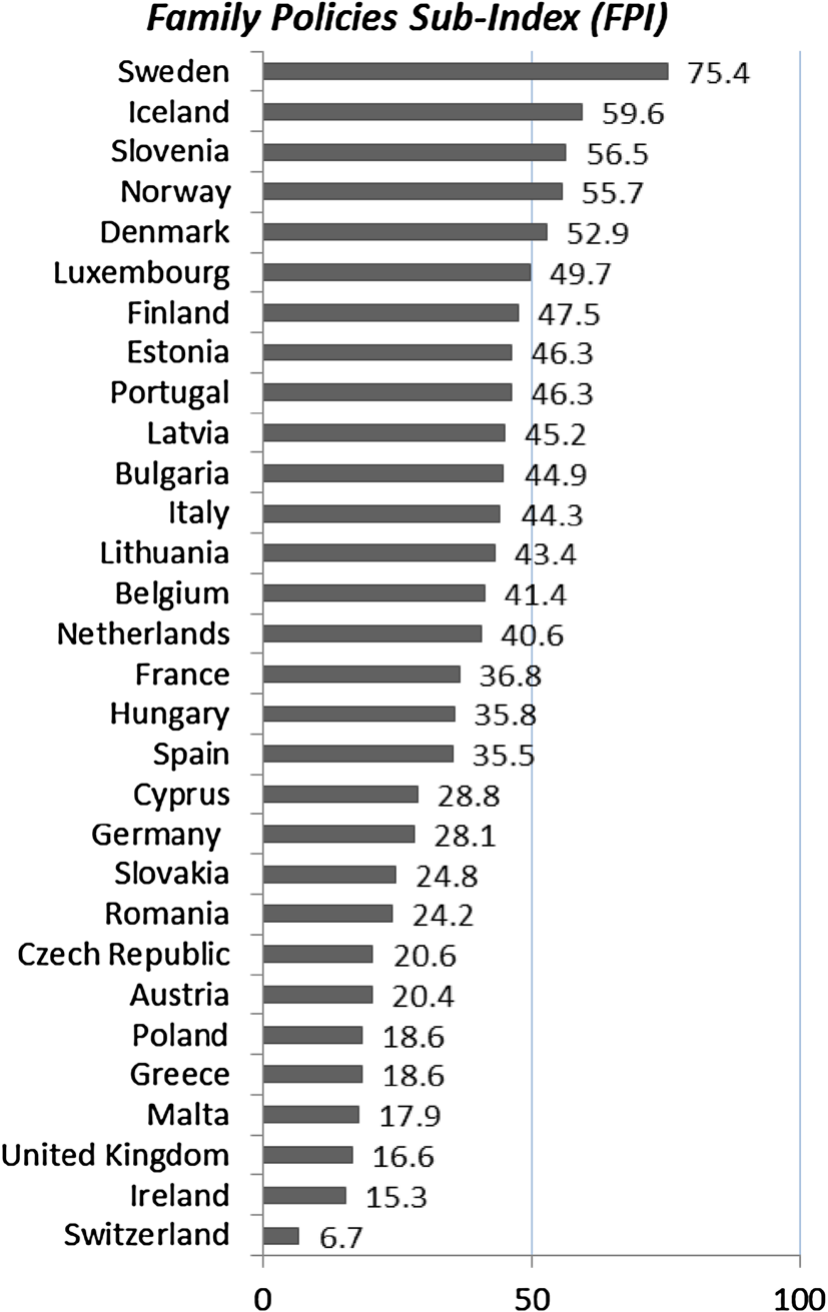
Family policies sub-index

**Fig. 4:**
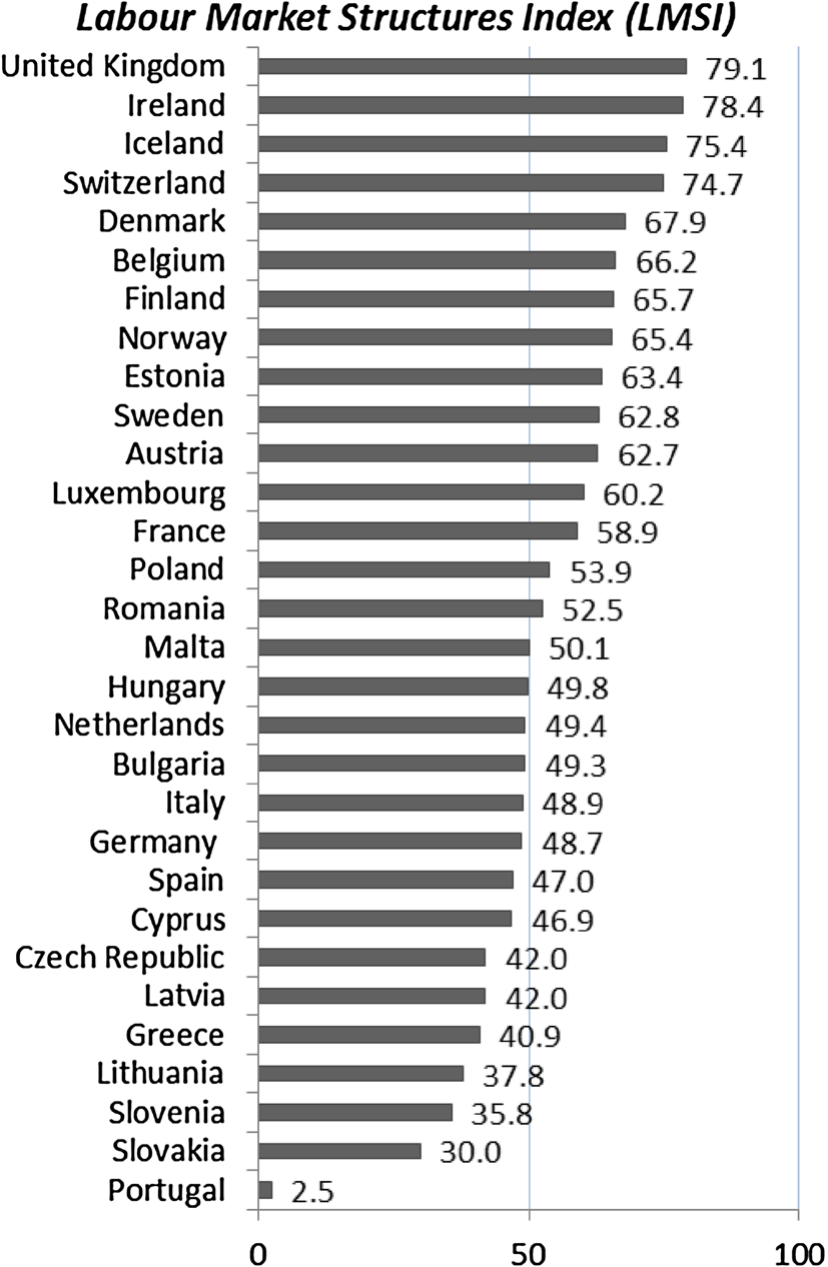
Labour market structures sub-index

**Fig. 5:**
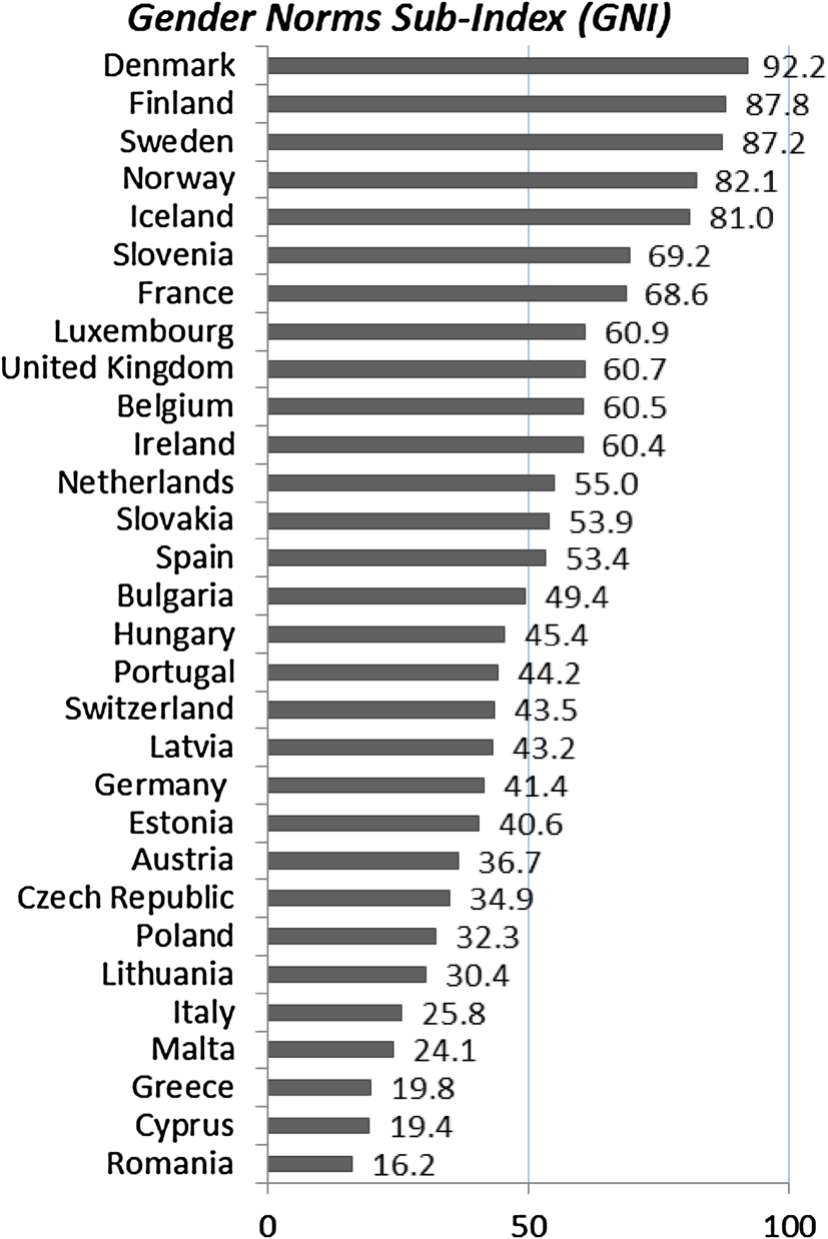
Gender norms sub-index

**Fig. 6:**
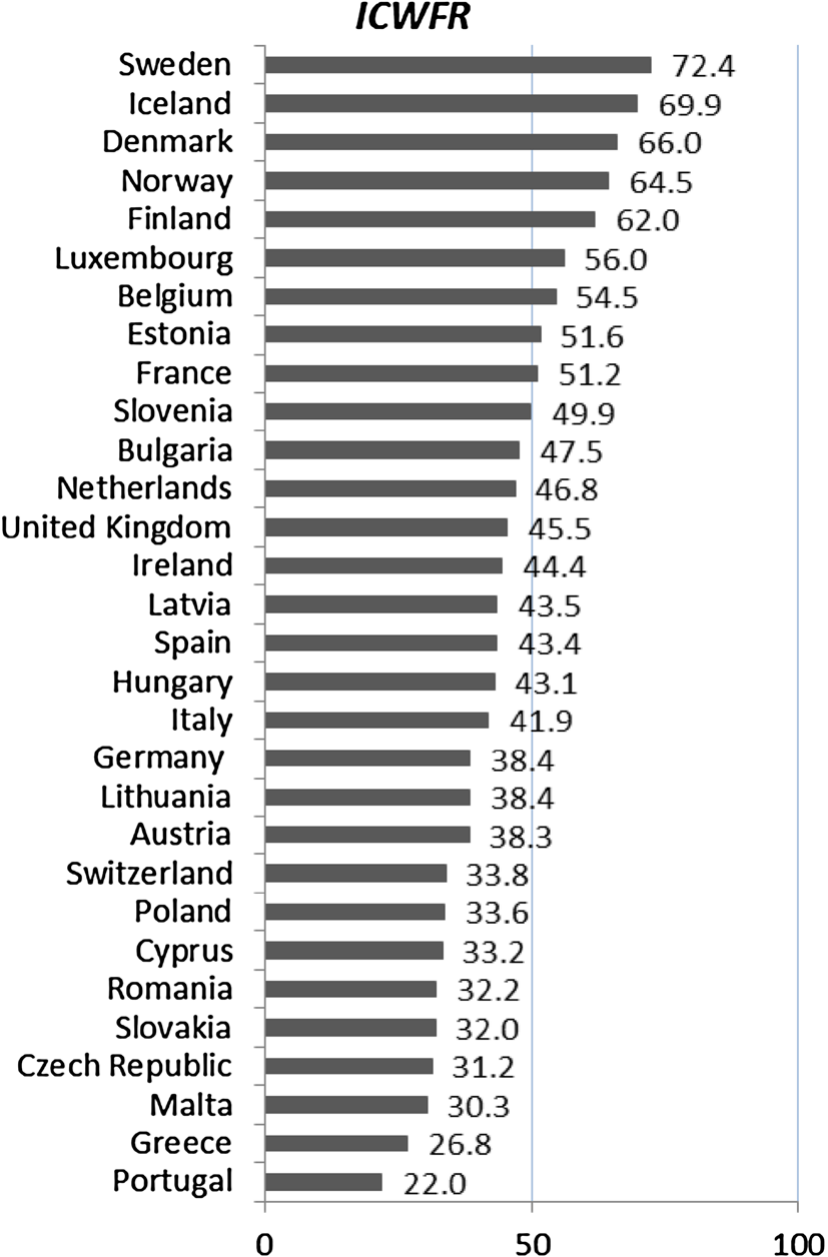
Index of conditions for work and family reconciliation

Following the operationalization scheme, we computed the ICWFR and the three sub-indices—FPI, LMSI and GNI—for 27 EU member states plus Iceland, Norway and Switzerland. The index scores are presented in Figs. 3, 4, 5 and 6 and in Table 6 (the scores with the associated standard errors).

**Table 6:** Index of conditions for work and family reconciliation (ICWFR) and its sub-indexes [family policy index (FPI), labour market structures index (LMSI) and gender norms index (GNI): scores, median simulated scores, scores ± SD

Country	Score				Median simulated score				Score ± SD			
	FPI	LMSI	GNI	ICWFR	FPI	LMSI	GNI	ICWFR	FPI	LMSI	GNI	ICWFR
Austria	20.4	62.7	36.7	38.3	21.9	62.8	36.9	39.6	(17.089; 23.768)	(62.528; 62.896)	(36.018; 37.283)	(35.1; 41.442)
Belgium	41.4	66.2	60.5	54.5	42.6	66.6	60.6	55.3	(38.695; 44.071)	(65.186; 67.132)	(60.41; 60.62)	(52.485; 56.519)
Bulgaria	44.9	49.3	49.4	47.5	46.2	51.7	50.0	49.1	(42.203; 47.682)	(43.818; 54.743)	(47.999; 50.773)	(43.972; 51.112)
Cyprus	28.8	46.9	19.4	33.2	31.1	49.4	19.7	35.5	(23.536; 34.033)	(43.97; 49.763)	(18.741; 20.002)	(29.535; 36.928)
Czech Republic	20.6	42.0	34.9	31.2	22.3	42.3	35.4	32.4	(16.76; 24.409)	(41.484; 42.526)	(33.881; 35.922)	(28.616; 33.853)
Denmark	52.9	67.9	92.2	66.0	55.0	68.0	92.2	67.1	(48.356; 57.518)	(67.639; 68.111)	(92.103; 92.254)	(63.371; 68.691)
Estonia	46.3	63.4	40.6	51.6	49.0	63.9	40.7	53.0	(40.384; 52.295)	(62.336; 64.528)	(40.305; 40.873)	(48.344; 54.844)
Finland	47.5	65.7	87.8	62.0	48.1	66.2	87.9	62.6	(46.322; 48.742)	(64.622; 66.809)	(87.57; 87.944)	(60.223; 63.774)
France	36.8	58.9	68.6	51.2	38.7	59.0	68.8	52.3	(32.328; 41.172)	(58.764; 59.129)	(68.201; 69)	(48.461; 53.854)
Germany	28.1	48.7	41.4	38.4	29.8	49.0	41.7	39.5	(24.335; 31.885)	(48.007; 49.43)	(40.741; 42.093)	(35.997; 40.878)
Greece	18.6	40.9	19.8	26.8	20.0	41.4	20.3	27.9	(15.187; 21.973)	(39.92; 41.906)	(18.576; 20.995)	(24.073; 29.433)
Hungary	35.8	49.8	45.4	43.1	37.2	51.5	45.9	44.5	(32.676; 38.928)	(45.961; 53.592)	(44.29; 46.513)	(39.934; 46.215)
Iceland	59.6	75.4	81.0	69.9	61.2	75.2	81.3	70.6	(55.884; 63.266)	(74.638; 76.114)	(80.414; 81.65)	(67.741; 72.056)
Ireland	15.3	78.4	60.4	44.4	16.3	79.1	60.5	46.3	(13.196; 17.455)	(76.949; 79.925)	(59.971; 60.778)	(39.648; 49.13)
Italy	44.3	48.9	25.8	41.9	46.2	49.1	26.6	43.0	(40.909; 47.726)	(48.48; 49.365)	(23.837; 27.679)	(39.606; 44.263)
Latvia	45.2	42.0	43.2	43.5	48.6	43.9	43.8	45.8	(41.437; 48.961)	(37.725; 46.239)	(42.003; 44.471)	(40.2; 46.816)
Lithuania	43.4	37.8	30.4	38.4	44.2	38.6	32.3	39.5	(41.417; 45.299)	(36.114; 39.549)	(26.229; 34.59)	(35.951; 40.856)
Luxembourg	49.7	60.2	60.9	56.0	52.9	60.4	61.2	57.5	(46.005; 53.37)	(59.974; 60.52)	(60.067; 61.663)	(54.205; 57.835)
Malta	17.9	50.1	24.1	30.3	21.4	52.2	25.6	33.4	(13.247; 22.548)	(48.237; 52.018)	(20.553; 27.61)	(26.093; 34.533)
Netherlands	40.6	49.4	55.0	46.8	41.1	49.5	55.1	47.1	(39.584; 41.675)	(48.915; 49.831)	(54.882; 55.174)	(46.009; 47.669)
Norway	55.7	65.4	82.1	64.5	56.1	65.6	82.1	64.8	(54.921; 56.562)	(64.9; 65.951)	(81.993; 82.112)	(63.504; 65.57)
Poland	18.6	53.9	32.3	33.6	20.6	54.9	34.1	35.7	(14.127; 23.037)	(51.614; 56.22)	(28.221; 36.386)	(28.875; 38.361)
Portugal	46.3	2.5	44.2	22.0	48.2	2.7	44.5	24.2	(42.005; 50.633)	(2.024; 3.001)	(43.452; 44.869)	(16.65; 27.256)
Romania	24.2	52.5	16.2	32.2	28.0	53.9	18.1	35.2	(19.183; 29.166)	(49.434; 55.573)	(12.127; 20.332)	(27.213; 37.103)
Slovakia	24.8	30.0	53.9	32.0	26.7	30.4	54.4	33.2	(20.686; 28.999)	(28.923; 31.035)	(52.877; 54.999)	(29.048; 34.857)
Slovenia	56.5	35.8	69.2	49.9	57.5	36.2	69.6	50.7	(54.3; 58.747)	(34.911; 36.655)	(68.352; 70.097)	(47.855; 51.947)
Spain	35.5	47.0	53.4	43.4	36.6	47.7	53.6	44.3	(33.084; 37.944)	(45.371; 48.7)	(53.089; 53.775)	(41.463; 45.366)
Sweden	75.4	62.8	87.2	72.4	75.5	62.9	87.2	72.6	(75.089; 75.704)	(62.504; 63.138)	(87.14; 87.292)	(71.644; 73.244)
Switzerland	6.7	74.7	43.5	33.8	7.5	83.0	43.9	37.3	(4.912; 8.463)	(70.551; 78.864)	(42.444; 44.521)	(27.73; 39.796)
UK	16.6	79.1	60.7	45.5	17.2	79.8	60.8	47.1	(15.064; 18.055)	(77.402; 80.701)	(60.518; 60.858)	(41.194; 49.721)

According to the FPI, the family policy-related CWFR are definitely best in Scandinavian countries and Slovenia, the lead being taken by Sweden (Fig. 3). This finding is consistent with the previous literature that consistently points out public policies in Scandinavian countries to be most supportive to work/family reconciliation (Korpi 2000; Gornick et al. 1997; Esping-Andersen 1999). The following positions (down to the middle of the ranking) are taken by several central and eastern European (CEE) countries (i.e. Estonia, Latvia and Bulgaria) which grant usually generous leave provisions for women and provide good childcare (Szelewa and Polakowski 2008; Kontula and Söderling 2008), Luxembourg (earned largely due to long and well-paid leaves for men), Finland, Portugal and Italy. Lithuania, Belgium, the Netherlands, France, Hungary and Spain are placed in the middle of the ranking. The remaining CEE countries (Hungary, Slovakia, the Czech Republic, Romania and finally Poland) are found in the lower part of the ranking, together with Austria and Germany. These CEE and the German-speaking countries are known in the literature to have more familialistic policies which support a traditional division of household labour (Szelewa and Polakowski 2008; Esping-Andersen 1999). The bottom of the ranking consists of Greece, Malta, the UK, Ireland and Switzerland, which are often pointed out as countries with a “residual welfare state” (Esping-Andersen 1999; Gornick et al. 1997). Interestingly, Italy and Spain do not perform that poorly in the ranking. Even though they are often described as unsupportive to work and family reconciliation (Gauthier 1996; Bettio and Plantenga 2004), they provide relatively good and cheap childcare for children aged 3+.

The UK and Ireland top the list regarding the labour market structures dimension (according to the LMSI). These two countries are known for having highly flexible labour markets with low employment protection which eases employment entry after a career break (Adserà 2004, 2005). Indeed, it is the weak employment protection legislation that earns these two countries their high position in the ranking—and not so much the availability of part-time jobs, which are rather badly paid there. The Nordic countries, Switzerland, Belgium and Estonia come after the UK and Ireland as employment protection legislation in these countries is only slightly stronger than in the English-speaking countries, and additionally employees in the Nordic countries have relatively high control over their work schedules. Austria, France, Luxembourg, some of the CEE countries (Poland, Romania, Hungary, Bulgaria), Malta, Italy, the Netherlands and Germany take the middle position in the ranking. In the German-speaking countries, France and Italy, employment protection legislation is stronger than in the above CEE countries, but they offer more opportunities for adjusting one’s working time to family obligations (wider possibilities to have flexible working hours or work part-time even when accounting for the relatively low quality of part-time jobs in the German-speaking countries). The bottom of the ranking is largely built by the remaining countries in southern Europe (Spain, Greece and Portugal) and the CEE area (Czech Republic, Latvia, Lithuania, Slovenia and Slovakia), all of them usually characterized by both strong employment protection legislation and rigid working hours.

Finally, the culture-related CWFR, as measured by the GNI, were found to be by far the best in the Nordic countries (Denmark, Sweden, Finland, Norway and Iceland), followed by Slovenia and France (Fig. 5). Next were Luxembourg, the UK, Ireland, Belgium, Slovakia, the Netherlands and Spain. The rest of the country ranking, according to the GNI, is occupied by the remaining post-socialist countries, the remaining southern European countries, as well as the German-speaking countries, all of which are known for displaying relatively traditional attitudes towards the division of household labour between women and men (Treas and Widmer 2000; Muszyńska 2007; Lück and Hofäcker 2003). Among these countries, culture-related conditions were found to be somewhat better in Bulgaria, Hungary, Portugal, Switzerland, Latvia and Germany; somewhat worse in Estonia, Poland, Austria, the Czech Republic, Lithuania, Italy and Malta; and by far the worst in Greece, Cyprus and Romania.

These three types of CWFR—i.e. family policy related, labour market related and culture related—describe the general setting for combining paid work and care, as measured by the ICWFR. Taking all three dimensions into account, this setting turns out to be unequivocally the best in the Nordic countries, with Sweden and Iceland in the lead. The Nordic countries are followed by Luxembourg, Belgium, three post-socialist countries (Estonia, Slovenia and Bulgaria), France and the Netherlands. The middle of the ranking is built by the Anglo-Saxon countries, followed by three more CEE countries (Latvia, Hungary and Lithuania) and two southern European countries, namely Spain and Italy. The German-speaking countries are positioned next. The worst CWFR are found in the remaining post-socialist countries (Czech Republic, Slovakia, Romania and Poland), Cyprus, and finally in Malta, Greece and Portugal.

### Results of the Uncertainty Analysis and the Sensitivity Analysis

The median simulated scores for the ICWFR, FPI, LMSI and GNI were very similar to the reference scores (see columns 6–9 in Table 6). Furthermore, the reference scores were always within an interval defined by the 5th and 95th percentile of simulated scores. This implies that the ICWFR and its sub-indices, despite having been computed with a normatively chosen imputation method and power of the generalized mean as well as with weights assigned to ensure balanced importance of the dimensions, components and sub-components, represent non-biased indicators of country-specific CWFR.

Among all the assumptions made, the weights assigned to the FPI, the LMSI and the GNI were the most influential on the ICWFR scores (see Table 7). They were responsible for 33, 16 and 5 % of the volatility in the ICWFR, respectively (without taking interactions between these weights and other weights, the imputation method or the power of the generalized mean into account). The remaining 40 % of the whole ICWFR variation is to be attributed to interactions between uncertainty factors, mostly between weights assigned to the FPI, LMSI and GNI. This means that normative choices related to the imputation method and the power of the generalized mean do not influence the stability of ICWFR scores, in contrast to weights.

**Table 7:** The first-order and the total effect measuring the contribution of selected uncertainty factors to the overall volatility in ICWFR, without (*S*_i_) and after accounting for interactions ($S_{{{\text{T}}_{\text{i}} }}$)

Input factor	ICWFR	
	First-order effect (*S*_i_)	Total effect ($S_{{{\text{T}}_{\text{i}} }}$)
Power of generalized mean	0.01	0.08
Imputation method	0.01	0.01
Weight attributed to FPI	0.36	0.74
Weight attributed to LMSI	0.16	0.49
Weight attributed to GNI	0.05	0.20
Sum	0.60	–

### Criterion Validity

As the CWFR are not directly observable, the concern with measurement quality of the ICWFR is critical. Following Alwin’s (2015) recommendations, we use criterion validity to test whether the translation of the theoretical features of the CWFR into the ICWFR ensures a good reflection of the concept. To this end, we examine whether the ICWFR correlates with fertility and indicators of mothers’ labour force participation, which constitute external criteria. These variables, as anticipated in the introduction, should correlate positively with the ICWFR because it is found that countries with better CWFR will score better in terms of fertility and labour force participation of mothers than countries with poor CWFR. Hence, a simple test for the criterion validity of the ICWFR is to correlate it with fertility and indicators of mothers’ labour force participation. Confirming a positive association gives indication for satisfactory validity of the index.

However, a two-way correlation analysis may not serve this purpose. Whereas countries with good CWFR are bound to display high fertility and high labour force participation of mothers and countries with poor CWFR will turn out poorly in both respects, countries with moderate CWFR may have moderate levels of fertility and mothers’ labour supply but may well display low fertility and high mothers’ labour supply or vice versa. For this reason, we prefer to look at the three-way relationships.

**Fig. 7:**
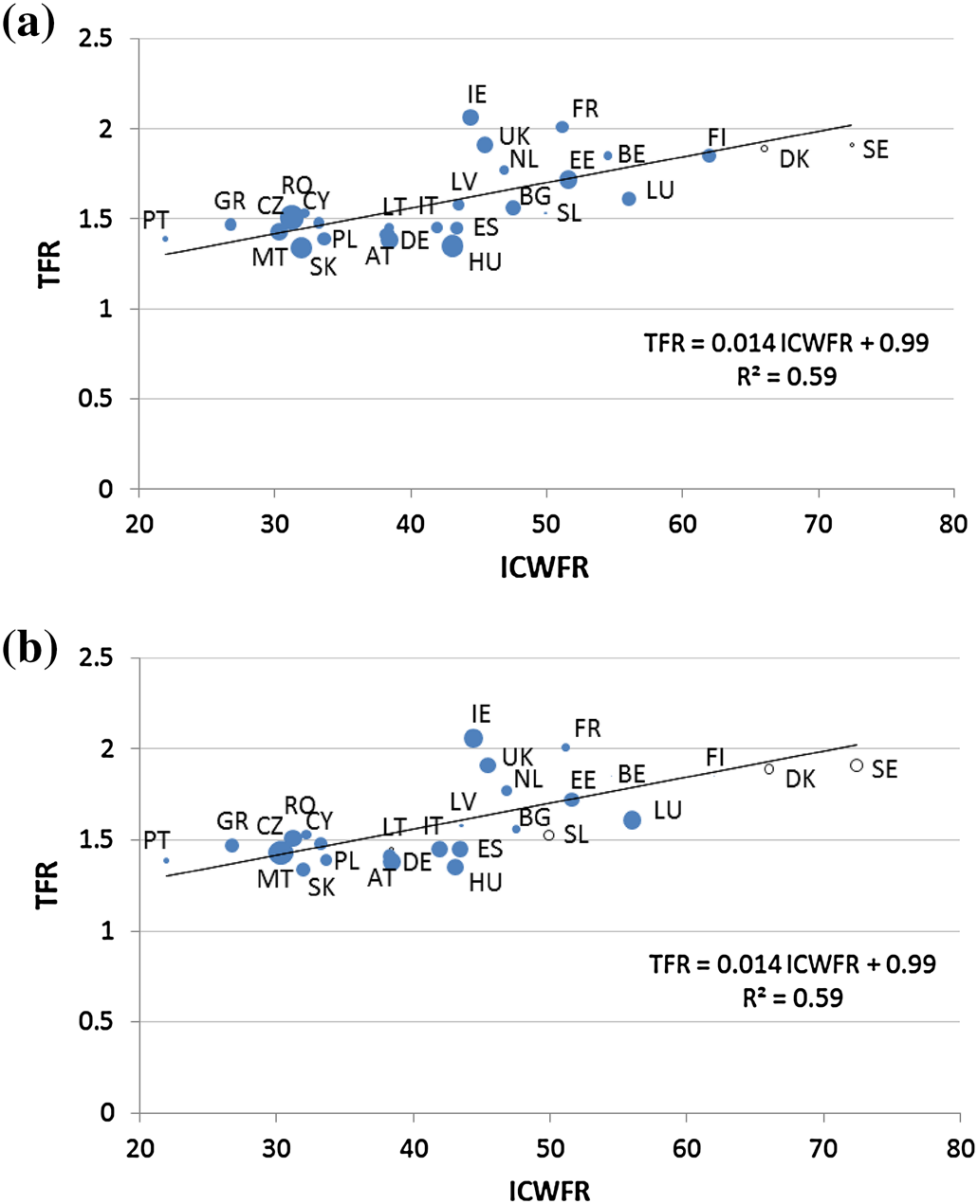
The index of conditions for work and family reconciliation (ICWFR) versus the total fertility rate (TFR) and **a** absolute difference between labour force participation rates of childless women aged 25–49 and mothers of children aged 0–6, **b** absolute difference between labour force participation rates of childless women aged 25–49 and mothers of two children, 2008. *Note* *Bubble area* is proportional to the absolute difference between employment rates of childless women and women with a 0- to 6-year-old child (aged 25–49). *Note* *Bubble area* is proportional to the absolute difference between employment rates of childless women and women with two children (aged 25–49)

Figure 7a, b displays bubble charts with the ICWFR on the *X*-axis, the period total fertility rate (TFR) on the *Y*-axis and two measures of mothers’ performance in the labour market expressed in the area of the bubbles, namely the absolute difference between the employment rates of childless women aged 25–44 and mothers of children aged 0–6 and the absolute difference between the employment rate of childless women aged 25–44 and women with two children, measuring the child-related employment penalties. These measures of mothers’ performance in the labour market were selected over a simple mothers’ employment rate since they take into account cross-country differences in general employment levels and thus better reflect the child-related employment penalties. All fertility and labour market indicators were measured in 2008, that is before the onset of the economic recession. In line with our expectations, the relationship between the TFR and ICWFR, marked by the regression line, is strong and positive. Furthermore, the bubbles tend to be larger in countries with less advantageous CWFR, which suggests that in these countries child-related employment penalties are particularly large. Ireland and the UK constitute special cases. They clearly deviate from the regression of TFR against the ICWFR, because fertility in these countries is much higher than what could be predicted on the basis of ICWFR. At the same time, child-related employment penalties in both countries are quite strong. Even though women in the UK and Ireland have relatively high fertility, the difficulties with combining paid work and care lead to strong child penalties.

## Discussion

In this paper, we proposed a conceptual scheme for country-specific CWFR and the ICWFR index that quantifies them, providing us with information about the magnitude of the barriers experienced by parents in combining work and care in a given country, as well as about the relative standing of a country regarding the conditions for work/family reconciliation. Additionally, the ICWFR allows us to perform a quantitative assessment of the associations between the CWFR and various aspects of individuals’ lives, including fertility, in a two-level regression framework. This assessment can be made with respect to the overall conditions or to their three dimensions, which means we are able to evaluate the relative importance of family policies, labour market structures and gender norms for childbearing and women’s performance in the labour market.

The ICWFR was computed for 30 countries: 27 EU member states plus Iceland, Norway and Switzerland, and describes the CWFR around the period of 2008–2010. It indicates that CWFR are unequivocally the best in the Nordic countries, followed by Luxembourg, Belgium, Estonia, France, Slovenia, Bulgaria and the Netherlands. These findings are in line with previous results cited in the literature, which have consistently indicated that in the Nordic countries, Belgium and France, public policies are the most supportive of working parents, and mothers’ involvement in the labour market is most widely socially accepted (Gauthier 1996; Gornick et al. 1997; Esping-Andersen 1999; Korpi 2000; Bettio and Plantenga 2004). The Netherlands has often been praised for wide opportunities to work part-time (Plantenga et al. 2012; Lewis et al. 2008), and Estonia, Bulgaria and Slovenia are the few post-socialist countries that remained supportive to mothers’ participation in the labour market after the fall of the communism (Szelewa and Polakowski 2008; Kontula and Söderling 2008; Koytcheva and Philipov 2008). Somewhat worse CWFR were found in the two Anglo-Saxon countries, which take a moderate position in the ranking. Despite a very low performance regarding the family policy dimension, Ireland and the UK defended their position thanks to very low levels of employment protection, which facilitates entry into the labour market after a career-related break (Adserà 2004, 2005), relatively flexible working hours and moderately modern gender norms. Italy and Spain together with three CEE countries—Latvia, Hungary and Lithuania—fell just behind, followed closely by the German-speaking countries. The bottom of the ranking is formed by four more post-socialist countries (Poland, Romania, Slovakia and the Czech Republic), which is consistent with previous findings by Szelewa and Polakowski (2008), and the remaining southern European countries (Cyprus, Malta, Greece and Portugal).

Even though the ranking provided by the ICWFR is largely consistent with what has already been known from the literature, it also provides new insights. First of all, it highlights a huge heterogeneity among the southern European countries, which are usually clustered together and considered as the least supportive towards work and family reconciliation. Second, it demonstrates that the CWFR in Germany and Austria not only fall behind those in Anglo-Saxon countries but are even a bit worse than in Italy or Spain. Third, it sheds more light on the situation in central and Eastern Europe, which is often neglected in the discussions on CWFR in Europe. It shows that post-socialist countries turn out quite diversely with respect to CWFR, Estonia, Slovenia and Bulgaria performing very well, while the Czech Republic, Poland, Slovakia and Romania are strongly unsupportive to working parents.

Until now, only few attempts at constructing a composite indicator of the CWFR have been made (Ray et al. 2010, Gornick et al. 1997, Matysiak 2011). Our ICWFR is more advanced than these previously developed indicators because (1) we take into account family policies, labour market structures and gender norms simultaneously; (2) we do not assume that shortages in one dimension of the CWFR can be fully compensated for by surpluses in another dimension; (3) we do not assume linear relationships between the overall index and its components, and in particular, our ICWFR is more sensitive to changes in those dimensions, components and sub-components that are largely unsupportive of work/family reconciliation; and (4) we take into account correlations between index dimensions while assigning aggregation weights. Finally, although the construction of the index required us to make some a priori assumptions about the compensation rate and aggregation weights, as previous authors have done as well, we demonstrated the robustness of the ICWFR to these assumptions in the uncertainty and sensitivity analyses we performed. The standard deviations of the ICWFR computed by uncertainty analysis for each country can be used as error terms in further analyses employing our index.

Despite these advances, the index we developed also has some limitations. First, the conceptual model of the ICWFR relies largely on the comprehensive literature review that included major theoretical approaches supplemented with most recent studies based on higher-quality data and applying more advanced methods. Although this research has been developing rapidly in recent years, it still fails to provide a number of important messages. Above all, it did not guide us in establishing the compensability rates as well as the importance of various family policies, elements of labour market structures and gender norms for determining the CWFR. In order to minimize the negative effects of these gaps in our knowledge, uncertainty and sensitivity analyses were performed to illustrate the sensitivity of our index to the assumptions we had to make.

Next, our index measures the average CWFR at the country level. This implies that the ICWFR assumes that all citizens of a country are affected by the CWFR equally, which may not always be true. For example, parental leave entitlements or eligibility for subsidized childcare may vary across social groups within a country. In some European countries, eligibility for social benefits and flexibility of work arrangements are determined by social partners and affect different sectors of the labour market differently (e.g. public vs. private sector).

Another drawback of our index is related to the choice of variables used for measuring certain sub-components of the CWFR. Facing limitations to data availability, in some cases we had to refer to variables describing outcomes rather than conditions of work and family reconciliation (e.g. proportion employed part-time, quality of part-time jobs or average number of hours spent in childcare). Nevertheless, the outcome variables we used were found to be correlated with conditions and we believe that the costs of dismissing certain sub-components from the analysis would exceed the cost of replacing the desired variables—measuring conditions—with the proxies we used. All in all, to the best of our knowledge the variables we used are the best indicators available in terms of relevance, validity, reliability, completeness and timeliness. The ICWFR can thus be used for assessing the magnitude of barriers faced by working parents across European countries and for analysing their effects on fertility. However, some caution is needed in the analyses of the effects of CWFR on women’s labour market outcomes, as some of the composite indicators used for constructing the ICWFR represent outcomes for, rather than conditions of, women’s employment, so the ICWFR should not be used for investigating the effects of CWFR on those outcomes (e.g. the extent and quality of women’s part-time employment).

Finally, being restricted by available data, we computed the ICWFR for just one time period and only for 27 EU member states plus Iceland, Norway and Switzerland. Undoubtedly, computing the ICWFR over a longer time period, for a larger number of countries or even for regions (NUTS 1/NUTS 2) within countries, would be very informative. Not only would it improve our knowledge on the variation in the CWFR over time and space, it could also enhance dynamic cross-country comparative analyses of the associations between the CWFR and fertility and women’s labour market outcomes, and could thus contribute to our knowledge of the importance of these conditions for fertility and women’s labour market outcomes on an even larger scale than is currently possible. As required data will become available on a wider scale in the future, computation of the ICWFR for other time periods, countries or regions within countries according to the methodology presented in this paper should be possible.

Against this background, our study has clear implications for future research. First, more research is needed to fill in the gaps in our knowledge, as noted above, on the importance of various aspects of CWFR in shaping fertility. Second, further improvements should be made regarding the availability of complete and reliable time series of CWFR indicators that refer not only to outcomes of work and family reconciliation but also to their underlying conditions, their international comparability and would allow us to compute the time series of ICWFR for a larger set of countries according to the methodology presented in this paper. This paper has shown which data are indispensable for measuring and monitoring CWFR over time and space as well as for further development of the research on how CWFR are shaping fertility.

## Footnotes

1 For examples of this method of establishing aggregation weights, see e.g. Athanasoglou et al. (2014), Węziak-Białowolska (2015).

2 Hot-deck imputation is a method for handling missing data in which each missing value is replaced with an observed response from a “similar” unit. The advantage of this method over the EM is that the hot-deck imputation belongs to the computationally simple yet effective imputation methods (Joenssen and Bankhofer 2012).

3 Details of the analysis are available upon request.
